# When Parents have a Severe Mental Illness : Can we Prevent Family Separation and Adverse Effects after Childbirth?

**DOI:** 10.1192/j.eurpsy.2022.93

**Published:** 2022-09-01

**Authors:** A.-L. Sutter-Dallay

**Affiliations:** CH Charles Perrens- PUPEA and Bordeaux University, Perinatal Psychiatry Network And Bphrc, Inserm Umr 1219, Bordeaux, France

**Keywords:** maternal psychiatric pathology, antenatal prevention, child protection, multidisciplinary work

## Abstract

This presentation will review the current state of knowledge about severe maternal perinatal mental illness. Severe disorders are associated with a higher prevalence of somatic difficulties during pregnancy, poorer quality of pregnancy follow-up and potential impairment of infant care. These children are therefore very vulnerable and require specific care. We will present how graduated care coordinated and above all integrated between psychiatry, obstetrics, neonatal pediatrics and child protection services allows for early and effective preventive interventions, both for the child’s development and maternal mental health. The concept of shared parenting will be particularly developed.

**Disclosure:**

No significant relationships.

